# Health from Public View-point

**Published:** 1919-03

**Authors:** 


### Health From a Public View-Point.

That the public is more interested in health problems from its viewpoint than ever before, and that physicians and surgeons are realizing that the people are vitally interested, was shown in the session of the Texas Conference of the American College of Surgeons held at Fort Worh on March 1. The keynote of the entire programme was "The American public has a right to be well," that subject being discussed ably by Dr. Charles B. Moulinier, president of the Catholic Hospital Association, Milwaukee, his paper being entitled, "Health a National Asset." More than a dozen addresses were delivered during the day dealing largely with hospital standardization, and kindred topics. At night the session was conducted in the big auditorium at Fort Worth and the public was invited to hear the addresses, which were couched in terms easily understood by the laity, that the laity might be more thoroughly aroused to the importance of proper hospital facilities. Among those who spoke was Dr. John A. Hornsby, of Washington, D. C., who had charge of the construction of the base hospitals in this country during the war. Dr. John G. Bowman, director of the organization, in a stirring address, called upon the public to share its right to be well. He said, among other things, "The hospital of the past has been a pleasant boarding house, but in the future it must be a radiating center for the good health of the community. What the school children wear and eat and the hygiene of the home are all subjects within the purview of the modern hospital." Other speakers were Dr. W. Burton Thornton of Houston, Dr. Howard R. Dudgeon of Waco, Dr. I. C. Chase of Fort Worth, Dr. Arthur C. Scott of Temple, Dr. Edward H. Cary of Dallas, Dr. Frank Boyd of Fort Worth, Dr. Charles Harris of Fort Worth, Dr. Witten B. Russ of San Antonio, Dr. L. F. Thompson of Fort Worth.

In the round table discussions most of those present took part. According to the daily papers, the following members of the Texas Committee on Standards were present: Dr. James E. Thompson, Galveston; Dr. Frank Cooke Beall, Fort Worth; Dr. W. Launcelot Brown, El Paso; Dr. Ira Carleton Chase, Fort Worth; Dr. Howard R. Dudgeon, Waco; Dr. John Thomas Moore, Houston; Dr. Arthur Carroll Scott, Temple; Dr. W. Burton Thorning, Houston, and Dr. Charles Scott, San Antonio.

In addition to a representative number of Fort Worth physicians, there were present, besides the committee mentioned, the following: Dr. James A. Hill, Houston; Dr. C. E. Collins, Waco; Drs. W. A. Duringer and A. M. McElbannon, Sherman; Drs. J. B. Franklin, Charles W. Flynn, Henry B. Decherd, L. L. VanZandt, H. M. Marks, C. M. Rosser and W. W. Samuel, Dallas; Drs. D. B. Leake and J. S. McClery, Temple; and Dr. C. F. Rose, Gainesville.

The meeting, it is thought, will stimulate the building of hospitals and the standardization of those already built in the State, and will therefore result in great good

